# Frankincense oil nanoemulsion induces selective cytotoxicity and over ROS-mediated oxidative stress and apoptotic DNA damage in Hep-G2 hepatic cancer cells

**DOI:** 10.1038/s41598-025-18201-9

**Published:** 2025-09-12

**Authors:** Hanan R. H. Mohamed, Esraa G. S. Ibrahim, Ahmed A. El-Sherif

**Affiliations:** 1 Department of Zoology, Faculty of Science, Cairo University, Giza, Egypt; 2 Department of Chemistry, Faculty of Science, Cairo University, Giza, Egypt

**Keywords:** Frankincense oil nanoemulsion, Hepatocellular carcinoma, Oxidative stress, DNA damage, *p53-mediated apoptosis*, Cancer therapy, Molecular biology, Biomarkers, Health care, Medical research

## Abstract

Frankincense oil nanoemulsion (FONE) exhibits high bioavailability, enhanced cellular uptake, and improved kinetic stability, making it a promising candidate for therapeutic applications. However, its potential effects on hepatocellular carcinoma (HCC) remain insufficiently explored. This study consequently investigated the cytotoxic and apoptotic activities of FONE on human hepatocellular carcinoma (Hep-G2) cells, focusing on cell viability, oxidative stress, DNA damage, and the regulation of apoptosis-related genes. Cell viability and proliferation were evaluated in Hep-G2 hepatic cancer cells and normal human skin fibroblasts (HSF) using the Sulforhodamine B (SRB) assay. Oxidative stress markers, DNA damage, and apoptotic gene expression were assessed through biochemical analysis, alkaline comet assay, and quantitative real-time PCR (qRT-PCR), respectively. Intracellular reactive oxygen species (ROS) levels were measured using 2,7-dichlorofluorescin diacetate (2,7-DCFH-DA) staining. Treatment with FONE at concentrations of 0.1, 1, 10, 100, and 1000 µg/ml for 48 h caused a significant, concentration-dependent reduction in Hep-G2 cell viability, with a half-maximal inhibitory concentration (IC50) of 176.14 µg/ml, while showing minimal cytotoxicity in HSF cells (IC50 = 515.7 µg/ml). Mechanistic investigations revealed that exposure to FONE IC50 concentration (176.14 µg/ml) significantly elevated ROS level, DNA damage, and lipid peroxidation (malondialdehyde level), accompanied by a marked decline in antioxidant defenses, including glutathione content and glutathione peroxidase activity. Gene expression analysis showed notable upregulation of pro-apoptotic genes *p53* and *Bax*, alongside a strong downregulation of the anti-apoptotic gene *Bcl-2*, in a concentration-dependent manner. FONE exerts selective cytotoxic effects against Hep-G2 cells, mediated by ROS-induced oxidative stress, DNA damage, and apoptosis induction. These findings highlight FONE’s potential as a targeted therapeutic agent for hepatocellular carcinoma. Further in vivo investigations and clinical evaluations are recommended to validate its efficacy and safety.

## Introduction

Hepatocellular carcinoma is one of the most prevalent forms of liver cancer, characterized by a notably high mortality rate. The prognosis for patients diagnosed with HCC can improve dramatically with early detection and accurate diagnostic procedures. The variety of treatment modalities available for HCC largely depends on the stage and severity of the disease. Key options include: liver transplantation, surgical resection, minimally-invasive techniques, targeted therapy and immunotherapy. The formulation of a personalized treatment plan is crucial for the effective management of this cancer^1^.

Despite advances in treatment, hepatocellular carcinoma presents considerable challenges within the field of oncology due to its elevated mortality rate and the limited nature of treatment options available. Therefore, assessing the cytotoxicity and genotoxicity of novel compounds in HCC cell lines is critical for determining their safety and potential therapeutic benefits^2,3^.

Recent advances in nanotechnology have led to the development of nanoemulsions with potential therapeutic applications. Nanoemulsions. thermodynamically stable colloidal systems composed of oil, water, and surfactants, have gained increasing attention in cancer therapy due to their ability to enhance the solubility, bioavailability, and targeted delivery of poorly water-soluble chemotherapeutic agents. These submicron-sized emulsions (typically < 200 nm) offer a stable and efficient platform for drug delivery, allowing for improved permeation and retention within tumor tissues via the enhanced permeability and retention effect^4^. In particular, nanoemulsion formulations have been shown to facilitate the controlled release of anticancer agents, minimize systemic toxicity, and enhance therapeutic efficacy. For example, curcumin-loaded nanoemulsions have demonstrated superior cytotoxicity and intracellular accumulation in breast and prostate cancer cells compared to free curcumin. Similarly, nanoemulsion-based delivery of docetaxel has shown enhanced anti-tumor activity and reduced side effects in models of lung and colon cancer. The incorporation of natural bioactive compounds into nanoemulsions, such as essential oils, has further expanded their utility in oncology. These natural compound-loaded nanoemulsions not only act as delivery vehicles but also contribute intrinsic anticancer activity through pro-apoptotic, anti-inflammatory, and oxidative stress-modulating mechanisms. As a result, nanoemulsions are increasingly recognized as promising tools for the development of safer, more effective, and more selective cancer therapies^5,6^.

Frankincense, an aromatic resin derived from Boswellia species, is known for its medicinal properties and has been utilized in traditional medicine for centuries due to its anti-inflammatory and anticancer properties^7^. Frankincense has been thus incorporated into nanoemulsion formulation to enhance its bioavailability, efficacy and targeted delivery of its bioactive compounds^8,9^. Recently, a nano emulsion of frankincense oil has shown promising effects in overcoming breast cancer resistance to the drugs paclitaxel and erucin. Nano emulsions are stable drug delivery systems that effectively transport hydrophobic drugs, addressing drug resistance issues.

This combination resulted in a sustained release and increased cytotoxicity of paclitaxel and erucin when delivered via the frankincense oil-loaded nano emulsion, compared to the drugs alone. It also significantly reduced oxidative stress, inflammation, and improved liver and kidney function, while restoring normal breast tissue architecture in a mouse model^9^. Additionally, the FONE demonstrated strong selective cytotoxicity against lung cancer A-549 cells by significantly upregulating pro-apoptotic genes (*DR5*,* FAAD*,* Caspase-8*,* p53*,* and Bax*) and downregulating anti-apoptotic genes (*Bcl2*,* NF-kB*,* and STAT-3*), leading to targeted apoptosis in these cells^8^.

These recently discovered promising findings alongside with the lack of studies on the therapeutic efficacy of FONE on hepatocellular carcinoma warrant investigation into the potential use of FONE in the treatment of hepatocellular carcinoma. Therefore, this study was undertaken to estimate the selective cytotoxic effects of FONE on Hep-G2 hepatocellular carcinoma cells in comparison to normal human skin fibroblasts (HSF). Specifically, the study focused on assessing its impact on cell viability, oxidative stress, genomic DNA integrity, and apoptosis induction in Hep-G2 cells.

## Materials and methods

### Chemicals

The FONE utilized in this study was sourced from Naqaa Company, located in Giza, Egypt. Biochemical kits were procured from Bio Diagnostic Company, present in Dokki, Giza, Egypt. All other chemicals employed throughout the research were obtained from Sigma-Aldrich Company (St. Louis, MO, USA), ensuring they were of high molecular grade to guarantee the reliability and precision of the data collected.

### Characterization of FONE

To ascertain the purity and nano-size of the FONE, imaging and examination of the nano droplets were conducted using a Transmission Electron Microscope (TEM). This analysis enabled the determination of the shape and average size of the frankincense nano droplets. Additionally, Fourier Transform Infrared Spectroscopy (FTIR) was performed by directing infrared light at the test sample, allowing for the observation of its chemical properties. Notably, any alterations in the absorption band pattern may signify structural changes in the material or the presence of contaminants^10^.

Furthermore, the distribution and Zeta potential of the FONE were assessed using a Zeta Sizer. This measurement is vital as it provides essential insights into the charge of particles within the formulation and their propensity to either aggregate or remain uniformly dispersed. Generally, particles with a Zeta potential exceeding + 30 mV or falling below − 30 mV are deemed stable, highlighting the significance of this parameter in nanoemulsion stability^10^.

### Cell seeding and quality control

Human hepatocellular carcinoma Hep-G2 and normal skin fibroblast (HSF) cell lines were procured from Nawah Scientific Inc., located in Mokatam, Cairo, Egypt. Both normal HSF and cancerous Hep-G2 cells were individually cultured in Dulbecco’s Modified Eagle’s Medium (DMEM: Gibco™, Thermo Fisher Scientific, Cat. No. 11965092), enhanced with 10% inactivated fetal bovine serum (Gibco™, Thermo Fisher Scientific, Cat. No. 10270106), 100 units/mL penicillin, and 100 mg/mL streptomycin (Pen-Strp: Gibco™, Thermo Fisher Scientific, Cat. No. 15140122). The cultures were maintained in an incubator at 37 °C with a 5% CO2 atmosphere^11^.

Prior to performing the Sulforhodamine B (SRB) cytotoxicity assay, both Hep-G2 and HSF cell lines were routinely screened for mycoplasma contamination using the MycoAlert™ Mycoplasma Detection Kit (Lonza, Switzerland), following the manufacturer’s instructions. Only mycoplasma-free cultures were used in the experiments. To ensure consistency and reduce biological variability, all assays were conducted using cells within the passage number range of 5 to 15.

### Determination of cell viability

Cells were harvested using 0.25% trypsin-EDTA (Gibco™, Cat. No. 25200056), counted using a hemocytometer, and re-suspended in complete DMEM medium at a density of 1 × 10³ cells/ml. Then, 100 µl (containing exactly 100 cells/well) was seeded in 96-well flat-bottom plates and incubated at 37 °C in a humidified 5% CO₂ atmosphere for 24 h to allow cell attachment and recovery. After incubation, cells were treated with FONE at five different concentrations (0.1, 1, 10, 100, and 1000 µg/ml) for 48 h. A vehicle control containing DMSO at a final concentration < 0.1% was included to match the solvent conditions of FONE treatments. The DMSO concentration used was confirmed in preliminary experiments to have no detectable effect on cell viability or morphology^12,13^.

Following treatment, cells were fixed with 10% (w/v) cold trichloroacetic acid (TCA) for 1 h at 4 °C, then washed three times with ice-cold PBS (defined as PBS stored at 4 °C and used immediately). After fixation, cells were stained for 10 min at room temperature in the dark with 0.4% SRB solution, freshly prepared in 1% acetic acid. Unbound dye was removed by washing three times with 1% acetic acid, and plates were left to air dry overnight. The protein-bound dye was solubilized in 200 µl of 10 mM Tris base (pH 10.5) per well. Absorbance was measured at 540 nm using a BMG LABTECH^®^ FLUOstar Omega microplate reader, model #: Omega-ACU-01. Cell viability was calculated relative to the untreated control. IC50 values were determined using nonlinear regression analysis in GraphPad Prism 9 based on three independent experiments, each performed in triplicate. The selectivity index (SI) was calculated using the formula: SI = IC50 (HSF) / IC50 (Hep-G2).

### Cell treatment

Hepatocellular carcinoma Hep-G2 cells were cultured under standard conditions in vented-cap T25 polystyrene flasks with tissue culture-treated surfaces(Corning^®^, Cat. No. 430639U). These cells were subsequently divided into two: untreated (control) and treated cells. Control cells were exposed to < 0.1% DMSO, which matched the solvent concentration used in treated samples and exhibited no toxicity on cell viability^13^. Treated Hep-G2 cells received either doxorubicin (prepared from a 2 mM stock solution in sterile distilled water and diluted to a final concentration of 2 µM) or FONE at concentrations corresponding to ¼ IC50, ½ IC50, and full IC50. Treatments were applied for 48 h. Following treatment, both control and treated cells were harvested, washed twice with ice-cold PBS, and stored at − 80 °C in PBS for subsequent molecular analyses. All experimental conditions were performed in triplicate to ensure accuracy and reproducibility.

### Estimation of Hep-G2 genomic DNA stability

The integrity of genomic DNA in Hep-G2 cells treated with doxorubicin and different concentrations of FONE was assessed using the alkaline Comet assay, following the protocols described by Tice et al.^14^ and Langie et al.^15^, with slight modifications. A 15 µl suspension of control or treated Hep-G2 cells was gently mixed with 60 µl of 0.5% low melting agarose prepared in PBS and maintained at 37 °C. This mixture was then layered onto clean microscope slides that had been pre-coated with a base layer of 1% normal melting agarose in PBS and allowed to solidify at 4 °C. After solidification, the slides were immersed in cold lysis buffer consisting of 2.5 M NaCl, 100 mM EDTA and 10 mM Tris, pH 10 with freshly added 10% DMSO and 1% Triton X-100. Slides were incubated in this lysis buffer at 4° C in the dark for 24 h. Following lysis, the slides were gently rinsed and placed in a freshly prepared alkaline electrophoresis buffer composed of 300 mM NaOH and 1 mM EDTA with a final pH > 13 and the slides were left in it for 15 min at 4° C to allow DNA unwinding. Electrophoresis was then carried out in a Cleaver Scientific horizontal electrophoresis unit (Model: HU25) at 25 V and 300 mA for 30 min, maintaining the temperature at 4° C. Upon completion of electrophoresis, slides were neutralized with 0.4 M Tris-HCl (pH 7.5), fixed with methanol, air-dried, and subsequently stained with 20 µg/ml ethidium bromide for 10 min in the dark at room temperature. The DNA comets were visualized using a ZEISS Axio Scope.A1 fluorescence microscope (Carl Zeiss Microscopy GmbH, Germany), and the extent of DNA damage was quantified using COMETSCORE™ Comet analysis software developed by TriTek Corp (http://www.autocomet.com/). The following parameters were calculated and reported as mean ± standard deviation (SD): Tail length, %DNA in tail and tail moment. These metrics were used to assess and compare the genotoxic effects of FONE with those of untreated control and doxorubicin-treated Hep-G2 cells.

### Evaluation of Hep-G2 ROS generation

Generation ROS level in Hep-G2 cells were assessed following 48-hour exposure to FONE, compared to untreated control and doxorubicin-treated cells, using 2,7-DCFH-DA staining, as described by Siddiqui et al.^16^. A 10 mM DCFH-DA stock solution was prepared in DMSO and stored at − 20 °C in the dark. The working solution (20 µM) was freshly diluted in serum-free medium immediately before use. Briefly, treated and control Hep-G2 cells were incubated with 20 µM DCFH-DA for 30 min at room temperature in the dark. During this period, the non-fluorescent dye penetrates the cells, where intracellular esterases cleave the acetate groups, and ROS oxidize the probe to yield the green-fluorescent product 2′,7′-dichlorofluorescein (DCF). After incubation, the cells were gently spread on clean glass slides and examined under an epi-fluorescence microscope (ZEISS Axio Scope.A1, Carl Zeiss Microscopy GmbH, Germany) using a FITC filter set (excitation: 488 nm; emission: 520 nm). Fluorescence images were captured at 200× magnification to visualize ROS-induced DCF fluorescence, which was used as an indicator of intracellular oxidative stress.

### Biochemical measurement of oxidative stress markers

Oxidative stress markers, including malondialdehyde (MDA), reduced glutathione (GSH), and glutathione peroxidase (Gpx), were measured in both untreated and treated Hep-G2 cells using kits provided by Biodiagnostic Chemical Company (Dokki, Giza, Egypt). The MDA level was determined based on its reaction with thiobarbituric acid at 95 °C in an acidic medium, forming a thiobarbituric acid reactive product. Cell suspension was mixed with the provided chromogen and incubated in boiling water path at 95 °C for 30 min. The absorbance of the resulting pink product was then measured spectrophotometrically at 534 nm^17^.

On the other hand, the GSH level was assessed based on the formation of a chromogen product generated by the reduction of Ellman’s reagent with GSH. To begin, equal volumes of cell suspension and trichloroacetic acid (TCA) were added in a microcentrifuge tube, gently mixed, and incubated for 15 min. at room temperature. Following incubation, the tubes were centrifuged at 3,000 rpm for 15 min. to separate the clear supernatant. Ellman’s reagent was then added to the supernatant, thoroughly mixed, and the absorbance of the resulting yellow chromogen was measured at 405 nm using a spectrophotometer^18^.

The activity of the antioxidant enzyme Gpx was assessed through a reaction where oxidized glutathione (GSSG) is produced upon the reduction of peroxides. The GSSG is subsequently recycled to its reduced form (GSH) by glutathione reductase, using NADPH as a cofactor. The decrease in NADPH absorbance at 340 nm indicates GPx activity. To perform this assay, 0.01 ml of cell suspension ml was added to micro-centrifuge tube containing 1.0 ml of assay buffer and 0.1 ml of NADPH reagent. The mixture was thoroughly mixed. Then 0.1 ml of hydrogen peroxide was added to initiate the reaction, the reaction, and the solution was mixed well. The reaction mixture was immediately transferred to a spectrophotometer, and the decrease in absorbance at 340 nm (A340/min) was recorded over a period of 3 min against deionized water blank. The oxidation of NADPH to NADP⁺ caused a reduction in absorbance at 340 nm, which served as a measure of GPx enzyme activity^19^. Results for oxidative stress markers were expressed as mean ± standard deviation (SD).

### Measuring the mRNA expression level of *p53*,* Bax* and *Bcl2* genes

To evaluate potential changes in the expression level of *p53* and *Bax* apoptotic genes and the anti-apoptotic *Bcl-2* gene in Hep-G2 cells treated with FONE, compared to untreated control and doxorubicin-treated cells, quantitative real-time PCR (qRT-PCR) was conducted. Total RNA was extracted using the GeneJET RNA Purification Kit (Thermo Fisher Scientific, USA) following the manufacturer’s protocol. The extracted RNA was then reverse transcribed into complementary DNA (cDNA) using the High-Capacity cDNA Reverse Transcription Kit (Applied Biosystems™, Cat. No. 4368814, Foster City, CA, USA). qRT-PCR was carried out using a StepOnePlus™ Real-Time PCR System (Applied Biosystems™, Model: 4376600) with SYBR™ Green chemistry. Each reaction had a final volume of 12 µL, containing: 1 µL cDNA template, 1 µL each of gene-specific forward and reverse primers sequences listed in Table 1^20,21^, 6 µL SYBR™ Green PCR Master Mix (Applied Biosystems™, USA), 3 µL nuclease-free distilled water. Thermal cycling conditions were as follows: Initial denaturation at 95 °C for 10 min, followed by 35 cycles of: Denaturation at 95 °C for 15 s, annealing/extension at 60 °C for 1 min, uniform for all primer sets. Gene expression level were normalized to the GAPDH housekeeping gene and analyzed using the comparative Ct (ΔΔCt) method. Results were expressed as mean ± standard deviation (SD) from three independent experiments.

**Table 1:** Sequences of the used primers in qRT-PCR.

Gene	Strand	Primer’s sequences
*BAX*	Forward	5’-CCGCCGTGGACACAGAC-3’
	Reverse	5’-CAGAAAACATGTCAGCTGCCA-3’
*BCL2*	Forward	5’-TCCGATCAGGAAGGCTAGAGT-3’
	Reverse	5’-TCGGTCTCCTAAAAGCAGGC-3’
*P53*	Forward	5’-CAGCCAAGTCTGTGACTTGCACGTAC-3’
	Reverse	5’-CTATGTCGAAAAGTGTTTCTGTCATC-3’
*GAPDH*	Forward	5’-GAAGGTGAAGGTCGGAGTCA-3’
	Reverse	5’-GAAGATGGTGATGGGATTTC-3’

### Statistical analysis

The results obtained from the alkaline Comet assay, oxidative stress markers and qRT-PCR were presented as mean ± SD and analyzed using the Statistical Package for the Social Sciences (SPSS) version 20. A one-way analysis of variance (ANOVA) followed by Duncan’s test was used to compare frankincense oil nanoemulsion-treated Hep-G2 cancer cells with control and doxorubicin treated Hep-G2 cells. Additionally, regression and correlation analyses were performed to evaluate the impact of different FONE concentrations on genomic DNA integrity, oxidative stress and gene expression in Hep-G2 cancer cells.

## Results

### Characterization of FONE

**Fig. 1:**
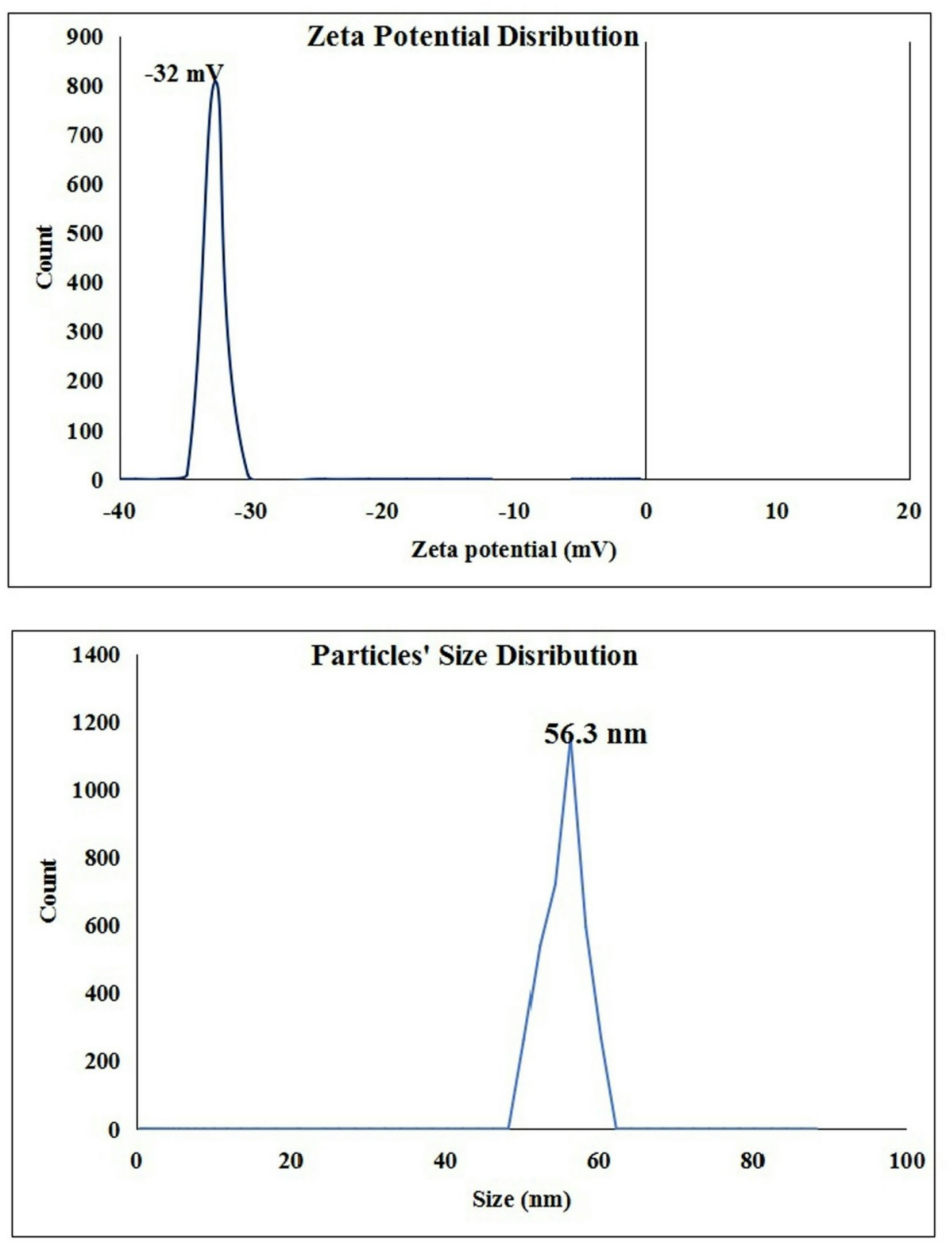
Zeta potential and particles’ size distribution of Frankincense oil nanoemulsion.

Measuring the Zeta potential value of FONE revealed the stability of Frankincense oil in its nano-formulation form. This stability was demonstrated by the detected Zeta potential value of -32 mV the FONE which was found in the stable region of Zeta potential value below − 30 mV as seen in Fig. 1. The Zetasizer analysis of the Frankincense oil nanodroplets distribution also confirmed the stability and well distribution of Frankincense oil nanodoplets through the measured average size of the Frankincense oil nanodroplets which is a high quality nanoemulsion with an average of 56.3 nm as displayed in Fig. 1.

**Fig. 2:**
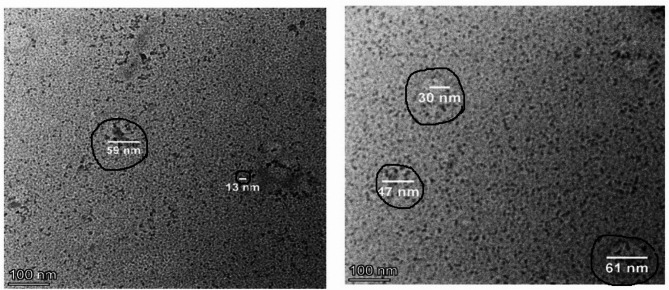
TEM imaging of Frankincense oil nanoemulsion.

**Fig. 3:**
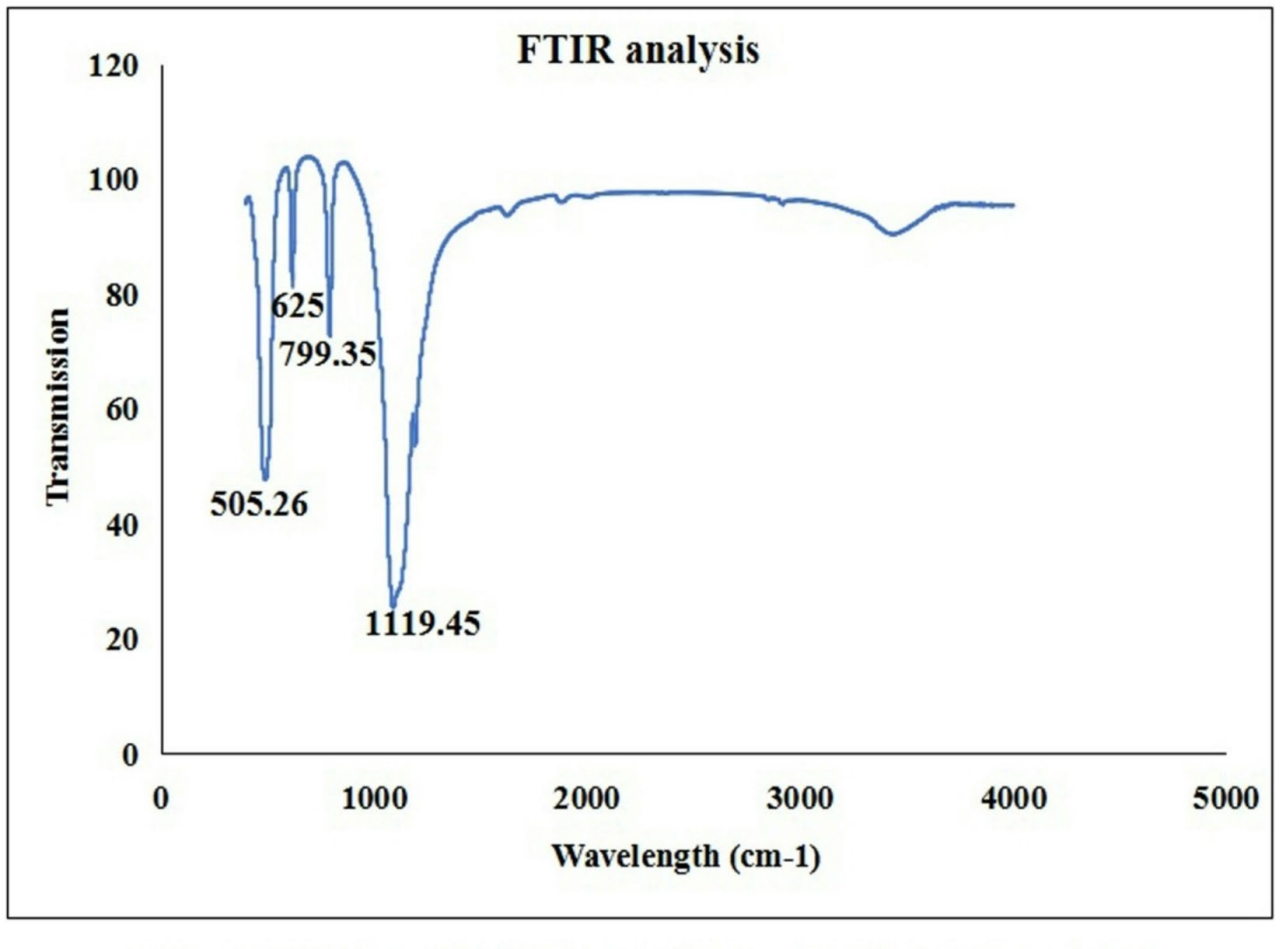
FTIR analysis of Frankincense oil nanoemulsion.

The imaging of the FONE using TEM demonstrated the well dispersion and spherical shape of Frankincense oil nanodroplets with an average droplets size of 42 nm (Fig. 2). The FTIR spectrum presented in Fig. 3 provides valuable insights into the chemical composition and structural properties of the Frankincense nanoemulsion through the absorption of infrared light by Frankincense nanoemulsion at specific peaks ranging from 4000 cm^−1^ to 399.19 cm^−1^ wavelength. These characteristic absorption bands arise from the vibrations of specific functional groups and molecular structures that define the chemical moieties within the Frankincense oil nanoemulsion. The fingerprint region, noticed from wavelength 1500 cm^−1^ to 500 cm^−1^ wavelength, exhibits a specific absorption band pattern unique to the FONE where the bands observed at wavelength 1119.45, 799.35, 625 and 505.26 cm-1 (Fig. 3) for example denote the hydroxyl and carbonyl groups along with hydrophilic interaction.

### Cell proliferation and viability

**Fig. 4:**
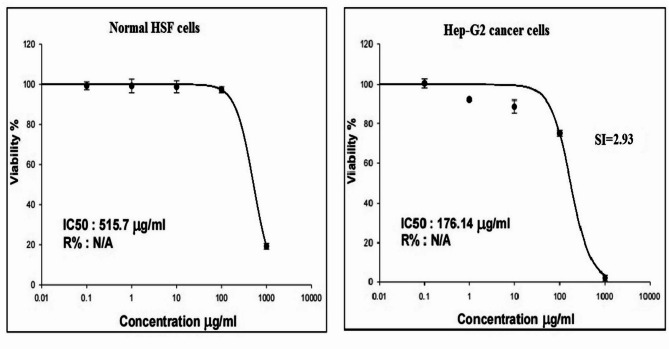
Viability of human normal HSF and hepatic cancer Hep-G2 cells after exposure to Frankincense oil nanoemulsions five concentrations (0.1, 1, 10, 100 and 1000 µg/ml) for 48 h. Results are expressed as mean ± SD. Triplicate was conducted per each concentration. SI: Selectivity index.

The results of the SRB cytotoxicity assay demonstrated a potent and selective cytotoxic effect of FONE against Hep-G2 hepatocellular carcinoma cells. Following 48-hour exposure to five concentrations of FONE (0.1, 1, 10, 100, and 1000 µg/ml), Hep-G2 cells showed a concentration-dependent decrease in viability, with a significant inhibition of proliferation. The IC50 value for Hep-G2 cells was calculated to be 176.14 ± 1.86 µg/ml (Fig. 4), indicating strong cytotoxic activity.

In contrast, normal HSF cells exhibited minimal cytotoxicity under the same treatment conditions. At 100 µg/ml of FONE, HSF cell viability remained high at 97.27 ± 1.53%, while a significant decrease was observed only at the highest tested concentration (1000 µg/ml), where viability dropped to 19.18 ± 1.44% (*p* < 0.05). The IC50 value for HSF cells was determined to be 515.7 ± 2.26 µg/ml, confirming a much lower sensitivity to FONE compared to Hep-G2 cells (Fig. 4). The calculated selectivity index was 2.93, further supporting the selective cytotoxicity of FONE toward Hep-G2 cells. To investigate the molecular mechanisms underlying this selective cytotoxicity, Hep-G2 cells were treated with concentrations corresponding to ¼ IC50 (44.03 µg/ml), ½ IC50 (88.07 µg/ml), and IC50 (176.14 µg/ml). The detailed findings of these molecular studies are presented in the following sections, with all data expressed as mean ± standard deviation (SD) from at least three independent experiments and accompanied by corresponding p-values to indicate statistical significance.

### Induction of genomic instability

Analysis of the alkaline comet assay results demonstrated significant, concentration-dependent genomic DNA damage in Hep-G2 cancer cells following 48-hour treatment with FONE at concentrations of 44.03, 88.07, and 176.14 µg /ml (Table 2). Compared to untreated control cells, FONE-Hep-G2 treated cells exhibited marked increases in key DNA damage parameters. Specifically, tail length significantly increased from 3.06 ± 0.62 μm in control cells to 24.31 ± 1.80 μm at 176.14 µg/ml FONE (*p* < 0.001); %DNA in tail markedly rose from 10.93 ± 1.77% to 38.29 ± 1.43% (*p* < 0.001); and tail moment significantly elevated from 0.37 ± 0.09 to 9.35 ± 0.68 (*p* < 0.001), all indicating substantial DNA fragmentation. In comparison, doxorubicin-treated cells showed a tail moment of 1.11 ± 0.04, which was significantly lower than that observed at the highest FONE concentration.

**Table 2:** Tail length, %DNA in tail and tail moment in the untreated control and treated hepatic cancer Hep-G2 cells with doxorubicin and frankincense nano-emulsion.

Treatment	Concentration	Tail length (px)	%DNA in tail	Tail moment (px)
Untreated cells	0.00	3.06 ± 0.62 ^a^	10.93 ± 1.77 ^a^	0.37 ± 0.09 ^a^
Doxorubicin	2 µM	7.99 ± 1.24 ^b^	13.77 ± 2.51^a^	1.11 ± 0.04 ^b^
Frankincense nano-emulsion	44.03 µg/ml	11.03 ± 1.78 ^b^	23.66 ± 1.64 ^b^	2.90 ± 0.61 ^c^
	88.07 µg/ml	16.81 ± 2.84 ^c^	26.16 ± 2.93 ^b^	4.51 ± 1.15 ^d^
	176.14 µg/ml	24.31 ± 1.80 ^c^	38.29 ± 1.43 ^c^	9.35 ± 0.68 ^e^
ANOVA		F = 61.58 *p* < 0.001	F = 77.88 *p* < 0.001	F = 88.45 *p* < 0.001

Results are expressed as mean ± S.

Results were analyzed using one-way analysis of variance followed by Duncan’s test to test the similarity between the control and treated Hep-G2 cancer cells.

Means with different letters indicates statistical significant difference at *p* < 0.001 between the compared cells in the same column.

**Fig. 5:**
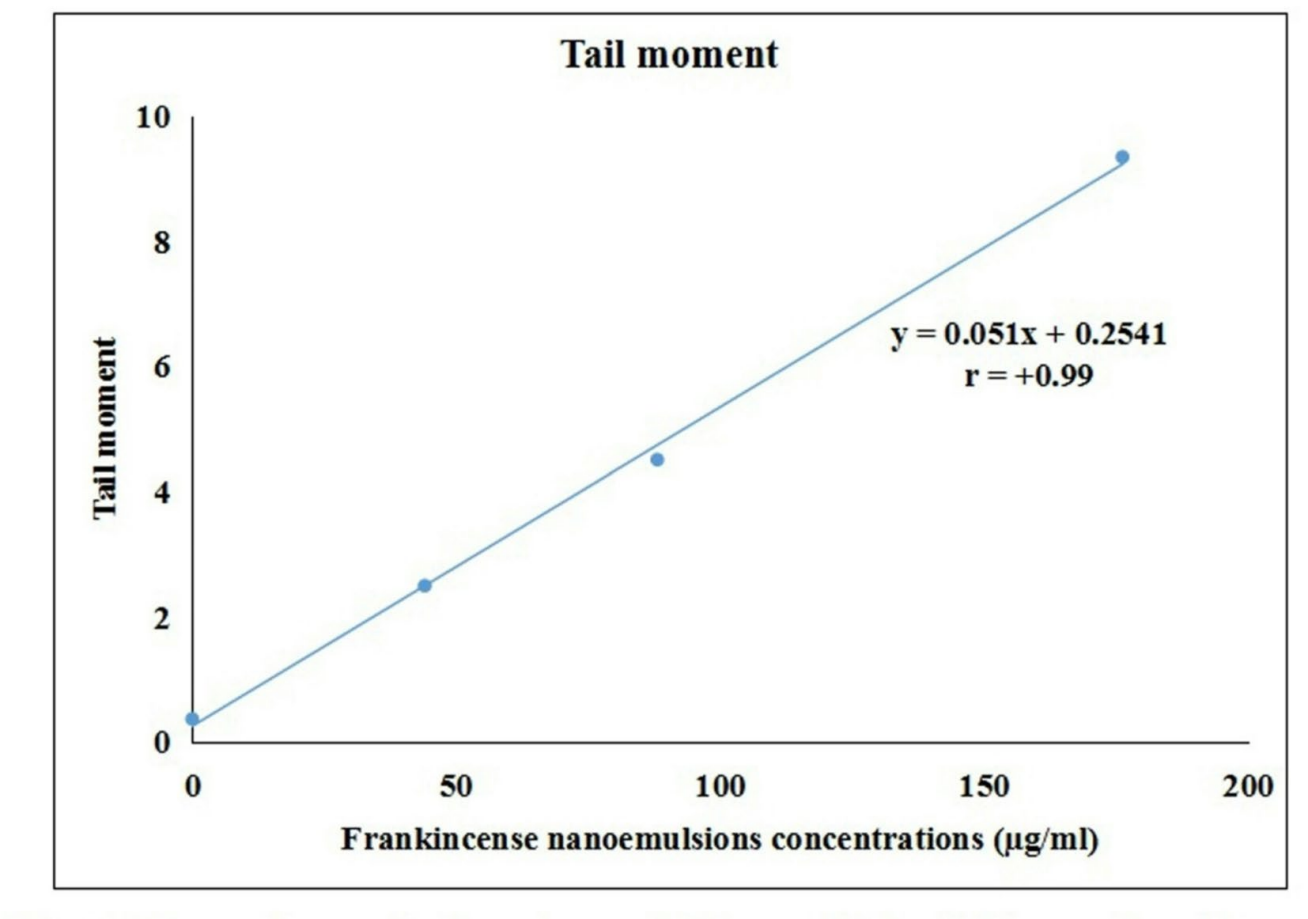
Regression analysis and correlation coefficient between Frankincense oil nanoemulsion concentrations and tail moment. Results are expressed as mean. Triplicate was done per each concentration.

**Fig. 6:**
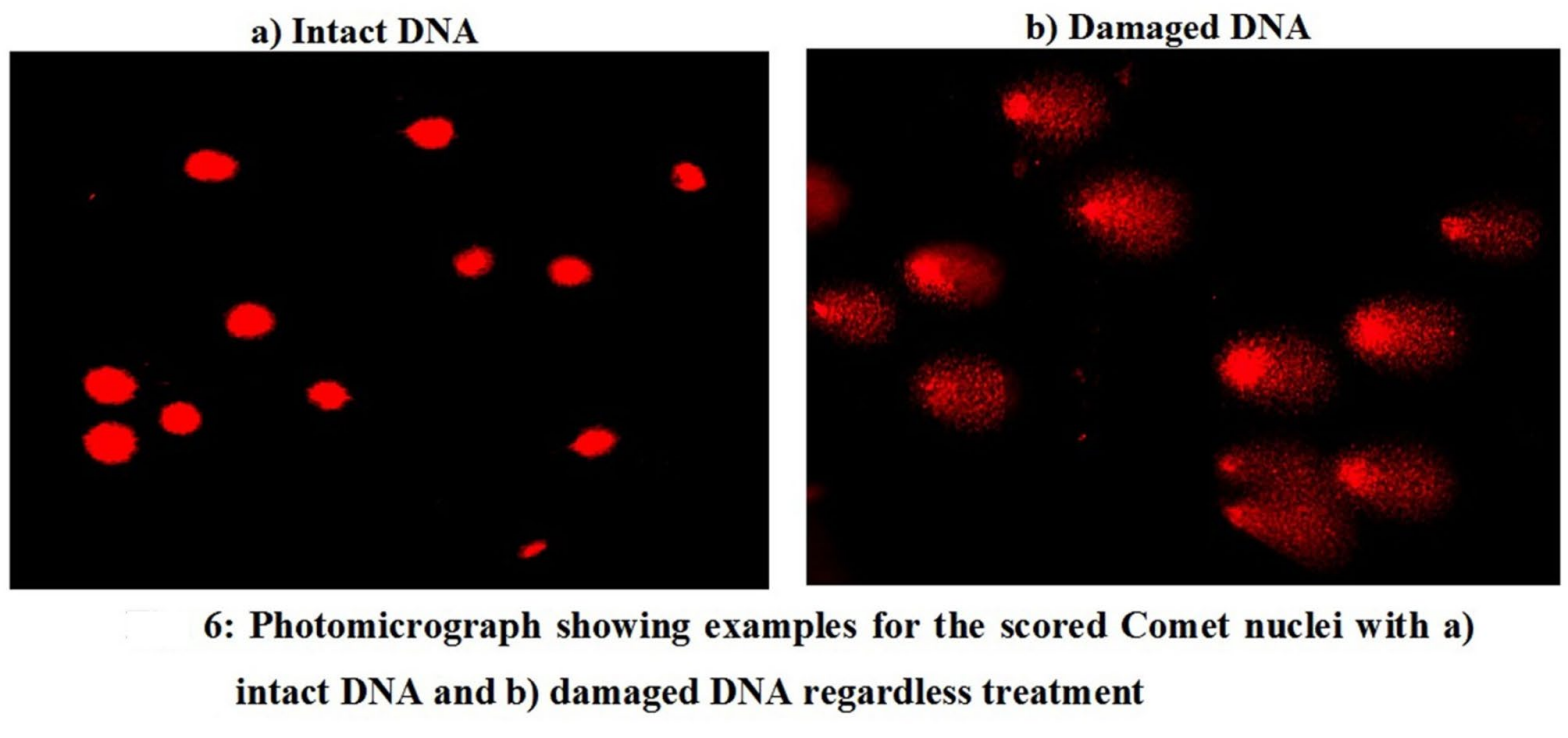
Photomicrograph showing examples for the scored Comet nuclei with (**a**) intact DNA and (**b**) damaged DNA regardless treatment. Magnification 200 X.

Furthermore, the DNA-damaging effect of FONE was strongly supported by a robust positive correlation (r = + 0.99) between tail moment and FONE concentration, as illustrated in the regression analysis (Fig. 5). Representative comet images showing the progression from intact nuclei to severely damaged DNA under increasing FONE concentrations are provided in Fig. 6, further confirming the genotoxic potential of FONE in Hep-G2 cells.

### Generation level of ROS

**Fig. 7:**
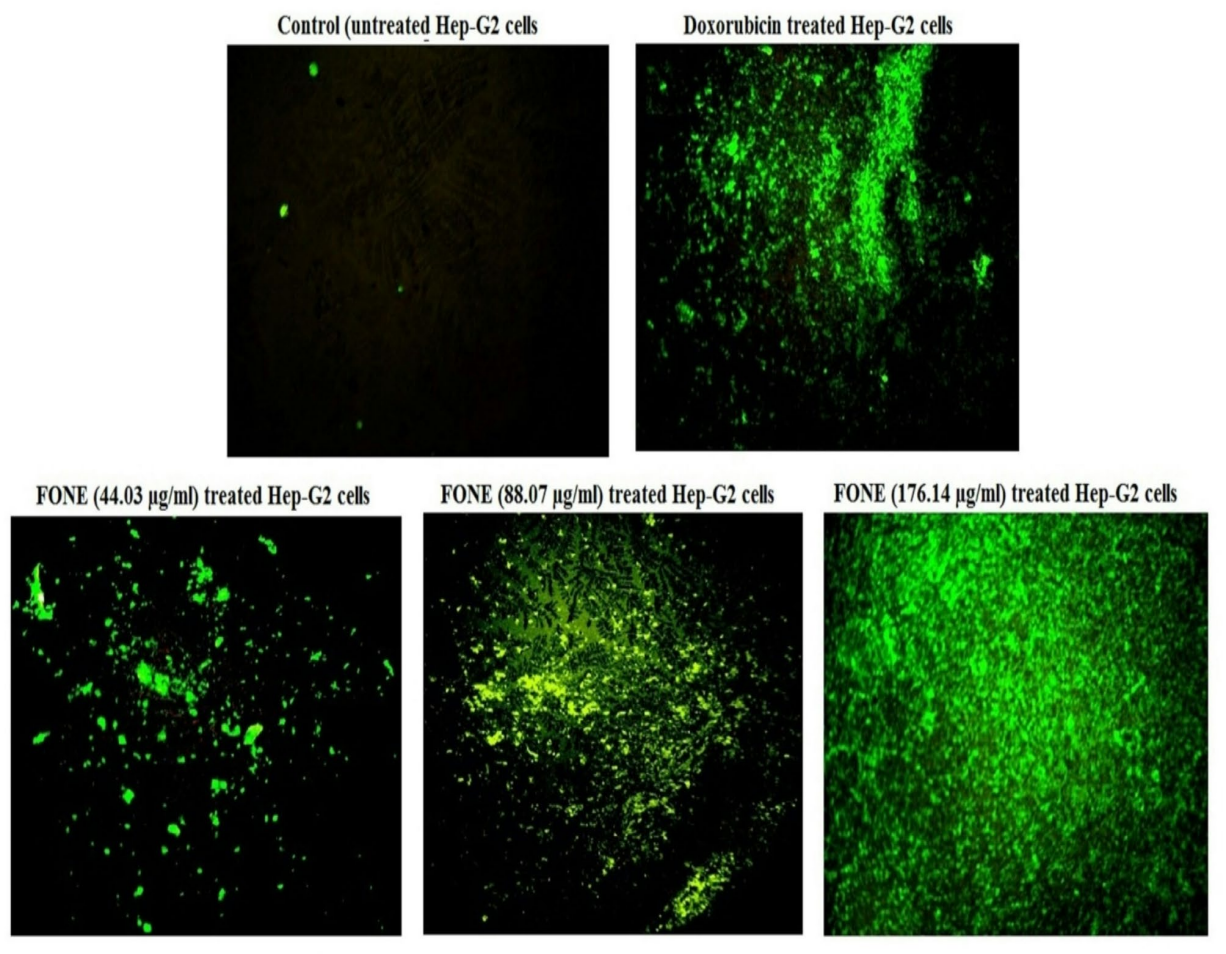
Level of ROS production within the control untreated and doxorubicin- or Frankincense oil nanoemulsion (FONE)- treated hepatic cancer Hep-G2 cells. Magnification 200 X.

Fluorescent staining of Hep-G2 cancer cells using 2′,7′-DCFH-DA revealed a marked, concentration-dependent increase in ROS generation following 48-hour treatment with FONE at concentrations of 44.03, 88.07, and 176.14 µg/ml. As shown in Fig. 7, this excessive ROS generation was evidenced by a marked rise in fluorescence intensity in FONE-Hep-G2 treated cells compared to both untreated control and doxorubicin-treated cells. The increase in fluorescence indicates elevated intracellular ROS level, supporting the oxidative stress-inducing effect of FONE in Hep-G2 cells.

### Oxidative stress induction

**Fig. 8:**
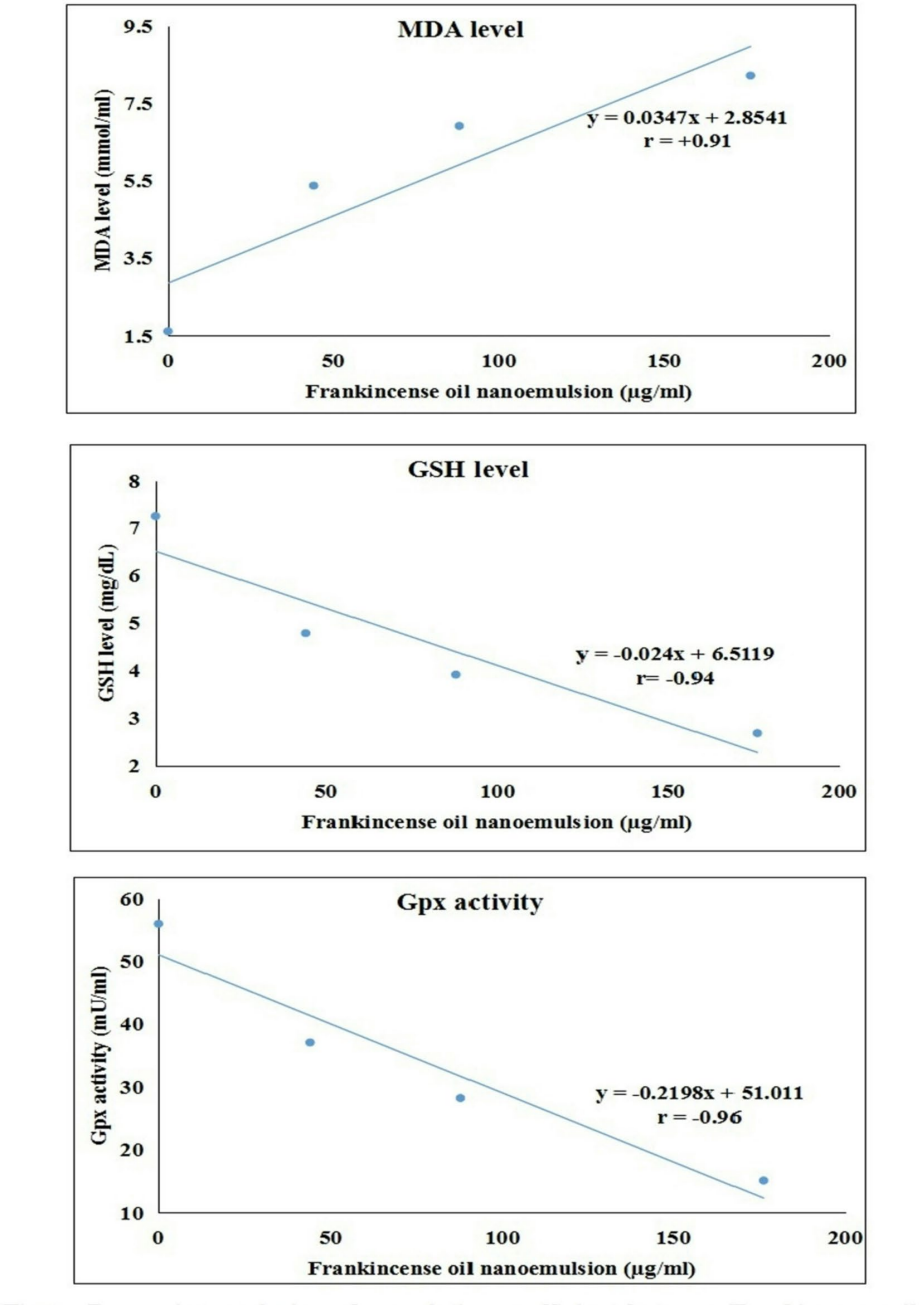
Regression analysis and correlation coefficient between Frankincense oil nanoemulsion concentrations and MDA level, GSH level and Gpx activity. Results are expressed as mean. Triplicate was done per each concentration.

Spectrophotometric analysis of oxidative stress markers revealed a pronounced, concentration-dependent induction of oxidative stress in Hep-G2 cancer cells after 48-hour treatment with FONE concentrations of 44.03, 88.07, and 176.14 µg /ml (Table 3).This was evidenced by a statistically significant increase (*p* < 0.001) in MDA level, rising from 1.61 ± 0.08 mmol/ml in untreated Hep-G2 cells to 8.21 ± 0.32 mmol/ml following treatment with the highest concentration of FONE (176.14 µg/ml). In parallel, a significant reduction (*p* < 0.001) in GSH levels was observed, decreasing from 7.25 ± 0.52 mg/dL in control Hep-G2 cells to 2.69 ± 0.42 mg/dL at 176.14 µg/ml FONE. Additionally, Gpx activity showed a marked decline (*p* < 0.001), dropping from 55.94 ± 2.13 mU/ml in untreated cells to 15.09 ± 2.48 mU/ml in Hep-G2 cells treated with the highest FONE concentration (176.14 µg/ml). Compared to doxorubicin-treated cells, FONE elicited more pronounced oxidative stress alterations across all parameters. The impact of FONE on oxidative stress was further supported by regression and correlation analysis (Fig. 8), which demonstrated a strong positive correlation between FONE tested concentrations and MDA level (r = + 0.91), as well as strong negative correlations between FONE concentration and both GSH level (*r* = − 0.94) and Gpx activity (*r* = − 0.96).

**Table 3:** Level of MDA and GSH and activity of Gpx enzyme in the untreated control and treated hepatic cancer Hep-G2 cells with doxorubicin and frankincense nano-emulsion.

Treatment	Concentration	MDA level (mmol/ml)	GSH level (mg/dL)	Gpx activity (mU/ml)
Untreated cells	0.00	1.61 ± 0.08 ^a^	7.25 ± 0.52 ^a^	55.94 ± 2.13 ^a^
Doxorubicin	2 µM	3.13 ± 0.41 ^b^	5.80 ± 0.56 ^b^	45.73 ± 3.72 ^b^
Frankincense nano-emulsion	44.03 µg/ml	5.37 ± 0.31 ^c^	4.79 ± 0.34 ^c^	37.06 ± 1.93 ^c^
	88.07 µg/ml	6.91 ± 0.35 ^d^	3.92 ± 0.20 ^d^	28.21 ± 2.53 ^d^
	176.14 µg/ml	8.21 ± 0.32 ^e^	2.69 ± 0.42 ^e^	15.09 ± 2.48 ^e^
ANOVA		F = 218.53 *p* < 0.001	F = 49.20 *p* < 0.001	F = 110.85 *p* < 0.001

Results are expressed as mean ± SD.

Results were analyzed using one-way analysis of variance followed by Duncan’s test to test the similarity between the control and treated Hep-G2 cancer cells.

Means with different letters indicates statistical significant difference at *p* < 0.001 between the compared cells in the same column.

### Expression of apoptotic and anti-apoptotic genes

As illustrated in Table 4, treatment of Hep-G2 cancer cells with FONE at concentrations of 44.03, 88.07, and 176.14 µg/ml for 48 h resulted in a statistically significant (*p* < 0.001) and concentration-dependent modulation of apoptosis-related gene expression. The expression of the pro-apoptotic gene *p53* significantly increased (*p* < 0.001) from 1.00 ± 0.00-fold in untreated control cells to 1.87 ± 0.11, 2.62 ± 0.20, and 3.49 ± 0.19-fold at the respective FONE concentrations of 44.03, 88.07, and 176.14 µg /ml, compared to 2.43 ± 0.13-fold in Doxorubicin-treated Hep-G2 cells. Similarly, *Bax* expression level markedly raised (*p* < 0.001) from 1.00 ± 0.00-fold in control Hep-G2 cells to 1.66 ± 0.07, 2.39 ± 0.06, and 2.16 ± 0.21-fold in Hep-G2 cells treated with the same FONE concentrations, exceeding the expression level (1.84 ± 0.10-fold) noticed in Doxorubicin-treated Hep-G2 cells. In contrast, expression of the anti-apoptotic gene *Bcl-2* was significantly downregulated (*p* < 0.001) in a concentration-dependent manner, decreasing from 1.00 ± 0.00-fold in control cells to 0.82 ± 0.05, 0.65 ± 0.04, and 0.49 ± 0.06-fold treatment with 44.03, 88.07, and 176.14 µg/ml FONE, respectively, compared to the downregulation (0.95 ± 0.06-fold) observed in Doxorubicin-treated Hep-G2 cells.

**Table 4:** Expression level of *p53*,* Bax and Bcl2* genes in the untreated control and treated hepatic cancer Hep-G2 cells with doxorubicin and frankincense nano-emulsion.

Treatment	Concentration	p53 gene	Bax gene	Bcl2 gene
Untreated cells	0.00	1.00 ± 0.00 ^a^	1.00 ± 0.00 ^a^	1.00 ± 0.00 ^a^
Doxorubicin	2 µM	2.43 ± 0.13 ^b^	1.84 ± 0.10 ^b^	0.95 ± 0.06 ^a^
Frankincense nano-emulsion	44.03 µg/ml	1.87 ± 0.11 ^c^	1.66 ± 0.07 ^b^	0.82 ± 0.05 ^b^
	88.07 µg/ml	2.62 ± 0.20 ^c^	2.39 ± 0.06 ^c^	0.65 ± 0.04 ^c^
	176.14 µg/ml	3.49 ± 0.19 ^d^	3.16 ± 0.21 ^d^	0.49 ± 0.06 ^d^
ANOVA		F = 117.03 *p* < 0.001	F = 161.48 *p* < 0.001	F = 52.65 *p* < 0.001

Results are expressed as mean ± SD.

Results were analyzed using one-way analysis of variance followed by Duncan’s test to test the similarity between the control and treated Hep-G2 cancer cells.

Means with different letters indicates statistical significant difference at *p* < 0.001 between the compared cells in the same column.

**Fig. 9:**
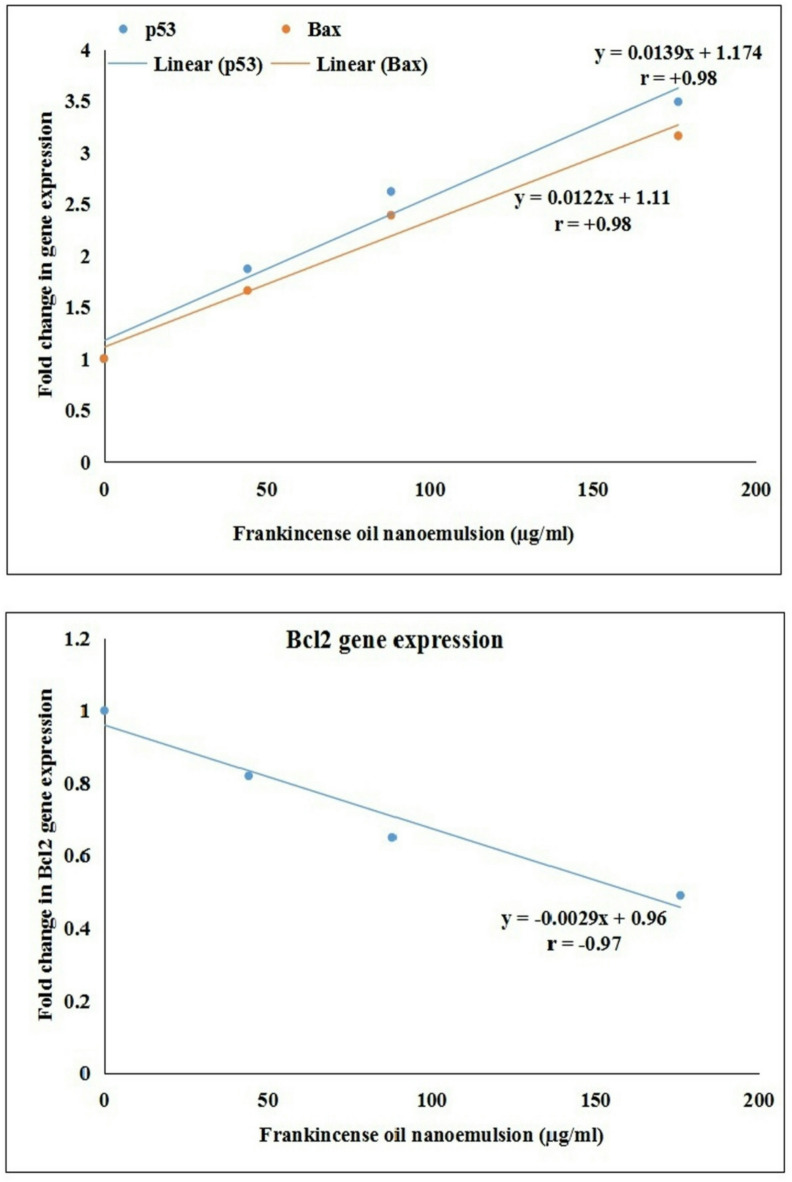
Regression anlysis and correlation coefficient between Frankincense oil nanoemulsion concentrations and fold change in the expression level of *p53*,* Bax* and *Bcl2* genes. Results are expressed as mean. Triplicate was done per each concentration.

Regression and correlation analysis further confirmed these findings, revealing strong positive correlations between FONE tested concentrations and the expression levels of *p53* and *Bax*(r = + 0.98 for both), and a strong negative correlation between FONE concentration and *Bcl-2* expression (*r* = − 0.97), as shown in Fig. 9. These results clearly demonstrate the concentration-dependent regulatory effect of FONE on apoptotic and anti-apoptotic gene expression in Hep-G2 cells.

## Discussion

Liver cancer, specifically hepatocellular carcinoma (HCC), is a significant global health issue ranking sixth in global incidence and fourth in cancer-related deaths. The prognosis for liver cancer remains poor, with a five-year survival rate below 9%. While surgical treatments offer potential cures, they are viable for only about 20% of patients. Chemotherapy options are limited, with sorafenib as the main drug providing benefits to only about 30% of patients, albeit with modest results and frequent development of drug resistance within six months. The challenges of hepatotoxicity, recurrence, and other adverse effects underscore the pressing need for alternative therapies^22^.

One promising avenue for liver cancer treatment lies in natural products, such as Frankincense derived from Boswellia tree resin. Rich in boswellic acids (BAs) with potential therapeutic properties, frankincense has a long history of medicinal use. These compounds target signaling pathways involved in tumor suppression, making them attractive candidates for cancer treatment. However, there is a notable gap in scientific data regarding the therapeutic effect of Frankincense oil, particularly in its nanoemulsion form, on hepatic cancer cells particularly its impact on cellular health and genetic integrity remains underexplored^23–25^.

The lack of comprehensive studies on the cytotoxicity and genotoxicity of FONE in hepatic cancer models limits our understanding of its potential as a therapeutic agent and raises important concerns about its safety and efficacy. This highlights the need for detailed investigations into the biological effects of FONE on hepatic cancer cells to better evaluate its potential for cancer treatment. To address this gap, the current study was conducted to assess the effect of FONE on the viability of hepatic cancer Hep-G2 cells and normal HSF cells, while also thoroughly examining its impact on genomic instability, oxidative stress, and apoptosis induction in Hep-G2 cells.

Screening cell viability and proliferation using SRB cytotoxicity assay revealed the strong and selective cytotoxicity of FONE against hepatic cancer Hep-G2 cells as manifested from the marked concentration-dependent reductions noticed in the percentage of Hep-G2 cell viability compared to the non-significant changes in the normal HSF cell viability. These results supported the findings of recent studies that highlighted the potent cytotoxic efficacy of FONE and Frankincense oil-loaded nanoemulsion formulations in both in vivo and in vitro models of human melanoma, lung, breast cancer and cervical cancer cell lines^8,26–28^.

The mechanisms by which Frankincense nanoemulsion induces cell death in cancer cells are complex involve multiple interconnected pathways and the precise mechanisms remain a subject of ongoing research. FONE may inhibit cancer cell proliferation and vitality through several potential signaling pathways, including the p53 signaling pathway and ROS-induced signaling pathway. One key proposed mechanism for its cancer cell death-inducing effects is the induction of oxidative stress. Nanoemulsions, such as FONE, can increase the production of ROS, highly reactive molecules that can damage cellular components and induce cell death. ROS-induced oxidative stress activates various pro-apoptotic signaling pathways, including p53, MAPK, and JNK pathways. Excessive ROS production can overwhelm cellular repair mechanisms, resulting in DNA damage and triggering apoptosis^6,7^. Induction of oxidative stress by FONE was manifested in this study through the significant elevations in ROS accumulation and MDA level along with significant decreases in the antioxidant GSH level and Gpx activity noticed within FONE treated Hep-G2 cells because excessive accumulation of ROS overwhelms the antioxidant defenses of Hep-G2 cancer cells and significantly damages its cellular components: lipids, proteins, and even DNA.

The highly reactive ROS are short-lived, oxygen-containing molecules attack and damage DNA forming various DNA lesions: single- and double-stranded DNA breaks, base modifications and cross links^29^. Consequently, FONE induced genomic DNA damage manifested in this study by the significant elevations in the DNA damage parameters measured in Hep-G2 cancer cells using alkaline Comet assay may result from the demonstrated high generation and accumulation of ROS within Hep-G2 cancer cells following exposure to FONE.

Oxidative stress and severe DNA damage can trigger apoptosis in cancer cells through various mechanisms. One proposed mechanism is the activation of apoptotic pathway via *p53-*mediated apoptosis. In many cancer cells, *p53* is either mutated or functionally impaired leading to defective apoptosis, however, in response to severe oxidative stress and DNA damage, even in cancer cells with mutated *p53*, alternative pathways can be activated. These pathways may involve the activation of other pro-apoptotic proteins or alterations in cellular signaling that still lead to apoptosis. The activation of pro-apoptotic proteins like *Bax* and *Bak* leads to mitochondrial outer membrane permeabilization, followed by the release of cytochrome C. This process activates the formation of the apoptosome and the caspase cascade, ultimately resulting in cell death^30,31^.

Oxidative stress and severe DNA damage have long been recognized as triggers for cancer cell apoptosis. A key pathway in this process involves *p53*-mediated apoptosis. When cancer cells experience oxidative stress, mutated *p53* is unable to induce the expression of antioxidant enzymes to combat the high levels of ROS. Instead, mutated *p53* upregulates the expression of cell proliferative and pro-apoptotic genes, leading to increased DNA damage and ultimately cell death^32,33^. Building on these findings, the FONE induced severe oxidative DNA damage discussed in this study, potentially triggering apoptosis in Hep-G2 cancer cells. This is evidenced by the concentration-dependent significant upregulation of apoptotic *p53* and *Bax* gene expression, as well as the marked downregulation of anti-apoptotic *Bcl2* gene expression observed in Hep-G2 cells following exposure to the FONE various concentrations. Overexpression of *p53*, in this context, induces apoptosis through the activation of other pro-apoptotic genes like *Bax* and the inhibition of the anti-apoptotic *Bcl2* gene, ultimately resulting in cancer cell death^34,35^.

## Conclusion

Overall, the findings of this study demonstrate that FONE exerts targeted cytotoxic effects on Hep-G2 hepatocellular carcinoma cells, with minimal toxicity toward normal HSF cells, indicating its potential for selective anti-cancer activity in vitro. The observed concentration-dependent induction of oxidative stress, DNA damage, and modulation of *p53*-mediated apoptotic gene expression suggests a possible mechanism underlying its cytotoxic effects. However, while these results provide valuable preliminary evidence, they are limited to in vitro conditions. Further investigations, including functional assays to confirm apoptosis and comprehensive *in vivo studies*, are essential to fully elucidate the therapeutic potential and safety profile of FONE. These future studies will be critical to determining whether FONE may contribute to the development of effective cancer treatment strategies.

## Data Availability

The datasets used and/or analyzed during the current study are available from the corresponding author on reasonable request.
